# FastGrow: on-the-fly growing and its application to DYRK1A

**DOI:** 10.1007/s10822-022-00469-y

**Published:** 2022-08-22

**Authors:** Patrick Penner, Virginie Martiny, Louis Bellmann, Florian Flachsenberg, Marcus Gastreich, Isabelle Theret, Christophe Meyer, Matthias Rarey

**Affiliations:** 1 ZBH - Center for Bioinformatics, Universität Hamburg, Bundesstr. 43, 20146 Hamburg, Germany; 2 Institut de Recherches Servier, 125 Chemin de Ronde, 78290 Croissy-sur-Seine, France; 3 BioSolveIT GmbH, An der Ziegelei 79, 53757 Sankt Augustin, Germany; 4 BioSolveIT GmbH, An der Ziegelei 79, 53757 Sankt Augustin, Germany

**Keywords:** Fragment-based drug design, Molecular shape, Fragment growing, Fragment evolution, Structure-based drug design, Molecular docking

## Abstract

Fragment-based drug design is an established routine approach in both experimental and computational spheres. Growing fragment hits into viable ligands has increasingly shifted into the spotlight. FastGrow is an application based on a shape search algorithm that addresses this challenge at high speeds of a few milliseconds per fragment. It further features a pharmacophoric interaction description, ensemble flexibility, as well as geometry optimization to become a fully fledged structure-based modeling tool. All features were evaluated in detail on a previously reported collection of fragment growing scenarios extracted from crystallographic data. FastGrow was also shown to perform competitively versus established docking software. A case study on the DYRK1A kinase, using recently reported new chemotypes, illustrates FastGrow’s features in practice and its ability to identify active fragments. FastGrow is freely available to the public as a web server at https://fastgrow.plus/ and is part of the SeeSAR 3D software package.

**Supplementary Information:**

The online version contains supplementary material available at 10.1007/s10822-022-00469-y.

## Introduction

Fragment-based drug design or discovery (FBDD) has become a mature paradigm in both the hit generation, as well as the lead optimization parts of early phase pharmaceutical research [1]. Three distinct pillars underpin FBDD techniques: fragment library design, fragment screening, and optimizing fragments into lead compounds, typically using linking, merging, and growing [2]. Computational methods have emerged to support each of these three areas specifically [3–6]. There is a special focus on the optimization of fragments to leads [7, 8], due to its methodological overlap with the general hit-to-lead optimization problem. A more general overview of FBDD and computational methods supporting FBDD can be found in dedicated reviews [2, 9, 10].

FBDD is frequently, if not almost exclusively, a structure-driven approach, meaning it relies on experimentally resolved or modeled structures of fragments that are bound to the target of interest. One popular method to progress from a low affinity fragment hit to a more traditional hit or lead is fragment growing [2, 11]. In fragment growing a molecule bound to a target is extended by attaching a suitable additional fragment. This is a common scenario in drug design that has inspired both academic developers as well as software suppliers to create specialized fragment growing software [12–15].

Structure-based fragment growing software is often based on docking methodology [8, 10, 13], which was initially developed with full-sized ligands in mind. Furthermore, there is very little consensus on how fragment growing should be validated. A few attempts have been made to standardize the validation of docking fragments into empty pockets [16, 17], but fragment growing validation is performed heterogeneously. This leads to a situation where it is unclear how appropriate and successful individual methodologies used for fragment growing actually are.

In this work we will describe our fragment growing workflow FastGrow based on the Ray Volume Matrix (RVM) shape descriptor [18], a pharmacophoric interaction description, and JAMDA geometry optimization [19]. Its features will be statistically evaluated on a previously reported data set of fragment growing steps extracted from crystallographic data [18] and compared to DOCK, a well-known, open source docking suite [20].

Furthermore, we will demonstrate FastGrow’s capabilities in the context of an FBDD campaign on the target DYRK1A (Dual Specificity Tyrosine-phosphorylation-regulated Kinase 1A). DYRK1A is a kinase implicated in various forms of cancer, neurodegenerative disease, and Down’s Syndrome. We will focus on the publications by Walmsley et al. [21] and Weber et al. [22].

## Methods

### FastGrow workflow

The FastGrow workflow is a combination of several recognizable or previously described features that in combination facilitate efficient structure-based fragment growing. Beginning at pose generation and scoring those poses with an empirical scoring function, FastGrow is also capable of searching with interaction constraints and built-in ensemble flexibility.

#### Ray volume matrix pose generation

FastGrow is primarily based on the Ray Volume Matrix (RVM) shape descriptor [18]. RVM shape screening is a fast way to generate accurate poses for thousands of fragments in a few seconds. In short, it uses a shape description symmetric to both pockets and fragments, to perform rapid comparisons and orient fragment conformations in a binding site. The input is a pre-calculated fragment database and a fragment bound to its target. The RVM is very fast and generates accurate poses, but it is limited to shape comparison.

#### JAMDA scoring and optimization

A remedy to this limitation is the inclusion of an interaction aware scoring function. Here we chose the JAMDA scoring function [19], an empirical scoring function that is modeled after well-known scoring functions such as PLANTS [23], ChemScore [24], and the original Böhm scoring function [25]. Thus, the JAMDA scoring function contains many similar score contribution terms. Its main novelty is its limited step length gradient-based optimization, which results in stable and consistent geometry optimizations.

In FastGrow JAMDA can perform the role of ranking fragments in the final output hit list and optimizing the poses that the RVM search produces, especially with respect to intermolecular interactions. JAMDA has several common interaction terms and can compensate for minor orientation errors in interacting groups with geometry optimization. FastGrow mostly performs restrained JAMDA geometry optimization. A restrained JAMDA geometry optimization tries to keep the position of the input core more or less the same as it optimizes the extensions. This is achieved with a quadratic penalty term that is applied if a core moves more than 0.5Å away from its input position.

#### Interaction constraints

A set of optional interaction constraints, which are modeled after generic pharmacophore features, can be used to guide the pose generation and filter fragments that cannot fulfill all interactions. The pharmacophore features can encode a number of types, most prominent of which are hydrogen bond donors, hydrogen bond acceptors, and hydrophobic points. Hydrophobic points refer to geometric points that abstract hydrophobic complementarity in a pharmacophoric way. Interaction constraints are represented by a point with a type and a tolerance radius. They will be referred to as search points. This is one of the most effective ways through which the user can directly interact with the workflow.

In the FastGrow web application, hydrogen bond acceptor and donor search points are generated according to interaction geometries defined by Nittinger et al. [26]. Hydrophobic search points are generated based on the definition in JAMDA [19], which is itself based on the ChemScore definition [24]. Search points are built from the predicted protein-ligand interactions and are placed on the ligand side of the interaction.

#### Ensemble flexibility

An input fragment can be positioned in multiple aligned binding sites so that it can be simultaneously screened against a database of fragments. This means multiple conformations of amino acid side chains and even backbone movements can be mimicked when generating the growing hits. When a fragment is scored against an ensemble only the best score of a fragment with respect to a member of the ensemble is used to position it on the hit list. This means that if a fragment conformation clashes with an amino acid side chain in three out of four conformations of the binding site, then the score of the fourth non-clashing, and by implication highest scoring, binding site will be used.

#### Feature validation

All statistical validation was done on either the cross-growing data set that was established in the previous paper on the RVM [18] or subsets of this data set selected for specific properties of the protein-ligand complexes.

The test cases of the cross-growing set simulate growing one ligand in a PDB structure using only the structural information of another, related PDB structure and its ligand. Both of these ligands are crystallized in the same binding sites, measured by sequence identity, and have a common core structure. The difference between them is one substituent/fragment with one single bond to the common core. The PDB structure of one ligand is used to create the test ligand by cutting it down to the common core and attaching the necessary fragment. The pose of the test ligand can then be compared to the reference crystal structure of that ligand, which has remained unused until this point. Around 300 such cases were generated for the PDBbind refined set v.2019 in the original publication, which contains more detail about the generation procedure [18].

The main evaluation metric used was the atom RMSD and whether it was above or below the conventional threshold of 2Å. Only the RMSD of the fragment atoms was measured. Confidence intervals were estimated by exploiting the binomial nature of a binary less than 2Å RMSD classifier, which can be approximated by a normal distribution. Further information on statistical methods can be found in Sect. 2 of the Supplementary Information. All feature specific validation test cases were compared to corresponding test cases in the cross-growing set.

#### Maintaining interactions

The real-world use case that was simulated by the interaction test cases is that a FastGrow user aims to maintain an important interaction when replacing or extending a substituent due to previous experience or external information. To this end, interactions were generated using the model discussed in the Interaction Constraints Sect. [19, 26] for the test cases of the cross-growing set. These were used as input and then regenerated later for the resulting pose. Interactions generated for the reference structure and the resulting pose were compared to see whether the input interactions could be maintained.

To ensure these interactions were stable across both binding site structures included in the cross-growing, search points were generated from interactions in the growing binding site and the reference binding site. The JAMDA/ChemScore [19, 24] definition of hydrophobic interactions is quite permissive and may lead to many hydrophobic search points. In validation we only consider fully hydrophobic rings and terminal hydrophobic groups. A search point that was generated in one binding site structure was considered stable if a search point of the same type could be generated in the other structure within 2Å of it. The generated search points were then available as an input to FastGrow. A comparison was then performed of growings of FastGrow without search point information, with search point information, and with additional restrained optimization. The resulting poses were compared with respect to how well they maintained the interactions by also generating search points for the grown poses. If the grown poses regenerated the search point within 2Å of the input search point, they had succeeded in maintaining the interaction.

#### Water replacement

In the second case that was used to validate interaction constraints, a user utilizes water molecules visible in crystallographic structures to either create new hydrogen bond interactions or to simply “push” a water out of a binding site. A subset of cross-growing test cases was extracted by checking whether a water was replaced in the course of the growing. This was detected by calculating van der Waals (vdW) radii overlaps between waters in the binding site that was used for growing and the ligand to be grown. If the ligand to be grown and a water exceeded a 60% vdW overlap threshold, the water was considered to have been replaced by the ligand to be grown. Search points were generated for replaced waters and the ligand. If a search point that was generated by a water molecule was within 2Å of a search point of the same type being generated by the ligand, then the search point of the water was used as a query for the water replacement growing. For the purposes of steric/hydrophobic water replacement, waters generated dummy hydrophobic interactions, in addition to the more physical hydrogen donor and acceptor interactions. The input for a water replacement was therefore the typical cross-growing input of a core in a binding site, as well as the search points generated by the replaced waters.

#### Handling binding site flexibility

To evaluate FastGrow’s ensemble flexibility implementation, we simulated a scenario where a user generates a set of representative binding site conformations and uses these to perform a growing. RMSD clustered ensembles of binding sites from the PDB were generated for all test cases of the cross-growing set using SIENA [27]. SIENA was run in the “docking” configuration, which implies, for example, binding site sequence identity. SIENA output was limited to five binding site conformations using the built-in all-atom clustering. The SIENA query binding site was the input binding site of the cross-growing test case in question, not the reference binding site. A minimum of two binding site conformations was necessary for a test case to be included in the ensemble flexibility subset. Note that the reference binding site containing the ligand to be grown is excluded from the binding site ensemble.

#### Comparison to docking

To establish FastGrow in the larger context of structure-based tools, it was compared to the well-known docking program DOCK [20]. DOCK is a high-profile [28] open-source suite of tools that has been validated in similar scenarios [20, 29]. The pose re-prediction capabilities of DOCK version 6.9 were compared to FastGrow using the cross-growing set in two configurations: a full flexible cross/re-docking of the ligand to be grown and a fixed anchor docking that receives the common core of the two ligands as an input, just as FastGrow does.

The protein-ligand complexes for docking were prepared in the same way as in the internal FastGrow workflow, which involves removing all crystal waters as well as molecules that clash with the input core from the binding site. Binding sites were re-protonated using protoss [30], which replaces the protonation scheme that is used by the PDBbind refined set. The active site for docking was defined as all atoms within 15Å of the native ligand. The binding site was chosen to be rather large so as to avoid any stability issues at its edges for the sphere generation. Spheres were generated by either the sphgen version included in the source code distribution or sphgen_cpp [31] when necessary. Those spheres within 10Å of the native ligand were selected for docking. Docking grids were generated using the GRID implementation that was provided with DOCK. Unless otherwise specified, all parameters were set to defaults originating either from the software package, DOCK publications [20, 29], and/or the DOCK fans mailing list [32]. The scripts that were used to automate this process are available at https://github.com/rareylab/dock_scripts. Only the actual call to DOCK after all input data had been pre-calculated was included in runtime measurements.

Anchored docking as implemented in DOCK handles input coordinates differently than FastGrow. FastGrow either freezes input coordinates or allows a minimal amount of movement in restrained JAMDA optimization. DOCK finds the largest rigid structure that is connected to a specified atom in the anchor. Unfortunately, as of DOCK 6.9 [33], it is not possible to rigidify structures manually, which means that in the worst case DOCK will still sample degrees of freedom in the input core. The atom specifying the anchor in our validation scenarios with anchored docking was always chosen to be the atom neighboring the linker atom. This was done to ensure the degrees of freedom of the growth vector were as comparable as possible to FastGrow. In the fragment growing enrichment case study the complete anchor was rigid, which meant that DOCK was not expected to sample any more degrees of freedom than FastGrow, and runtime comparison could be performed fairly.

#### DYRK1A case study

The first part of the DYRK1A case study simulated a fragment hit optimization from a micromolar fragment (PDB code: 7A4R) to a nanomolar ligand (PDB code: 7A5N). Three fragment growings were performed that roughly corresponded to three areas of optimization in the publication by Walmsley et al. [21]. The three fragment growings were performed with FastGrow and with DOCK by full re-docking, as well as anchored docking. The generated poses were compared and discussed.

The second part of the DYRK1A case study described screening libraries of fragments with FastGrow. The idea of this enrichment study was to measure how well FastGrow could pick out fragments known to be very active from a collection of generic fragments. Multiple high affinity ligands produced by collaborators at Vernalis and Servier contain a 2,6-diaminopyridine moiety. The diaminopyridine fragment from PDB code: 7AJW was extended by a collection of known active fragments generated from Servier/Vernalis ligands, as well as a collection of property matched generic fragments created by BioSolveIT [34].

Three workflows were used for growing: FastGrow’s pose scoring, FastGrow with restrained JAMDA optimization, and anchored docking with DOCK. All methods had to generate a pose for all fragments before these poses could then be scored. The workflow was designed to measure both the quality of the pose generation, as well as the screening power. Only fragments for which all methods could generate a pose were included in the statistics. After all fragments had been scored, the enrichment of all workflows was first compared to the enrichment achievable by sorting with “Rule of Three” properties [35], which served as the baseline, and then to each other. Confidence intervals were calculated using bootstrap re-sampling similar to the procedure presented in Stein et al. [36]. Further information on statistical methods can be found in Sect. 2 of the Supplementary Information.

## Results

The cross-growing set was regenerated on the PDBbind refined set v.2020 [37]. The procedure was the same as previously described [18], but resulted in more test cases due to the growth of the PDBbind. The previous cross-growing set contained 326 test cases and 155 unique fragments, whereas the new cross-growing set contained 425 test cases and 176 unique fragments.

The shape-based growing has improved slightly since the original publication [18] and recreated $66.8\pm 4.5\%$ (95% confidence interval) of ligand poses at an RMSD less than 2Å. Restrained JAMDA optimization at $70.8\pm 4.3\%$ showed minor improvements in RMSD (Fig. S1). As shown in the section below, the changes it performed seemed to be more beneficial to individual interaction geometries than to the pose as a whole.

The small position changes a restrained JAMDA optimization performed at the core atoms occasionally led to slightly higher RMSD. JAMDA was used in a restrained configuration that applied a quadratic penalty term to movement of the core atom. These are assumed to already be in the correct position, but a certain amount of movement was necessary for correct optimization. A full run of the cross-growing set took around half an hour without JAMDA optimization and three and a half hours with optimization, as can be seen in Table 1.

**Table 1:** Overview of the performance and runtime of FastGrow on the cross-growing set

	Success rate	Runtime [h]	Runtime per fragment [ms]
Growing	$66.8\pm 4.5\%$	0:31	24
Optimized growing	$70.8\pm 4.3\%$	3:16	158

FastGrow screens every unique fragment in the cross-growing set against every test case, which means the runtime per fragment is the full runtime first divided by 425 test cases and second divided by 176 unique fragments. Machine specifications can be found in Sect. 1 of the Supplementary Information

### Maintaining interactions

Search points were generated for all 425 test cases of the cross-growing set. 252 of these test cases generated at least one interaction that was stable across both binding site conformations. 85 acceptor search points and 21 donor search points were found in the cross-growing set. There were almost five times as many hydrophobic search points (a total of 532), than there were donor and acceptor search points, even though only terminal hydrophobic groups and “fully hydrophobic” rings were included. This was largely due to the very permissive definition according to ChemScore [24] in comparison to the geometrically constrained hydrogen bonds. The largest contributors to the number of hydrophobic search points were hydrophobic ring systems.

**Fig. 1:**
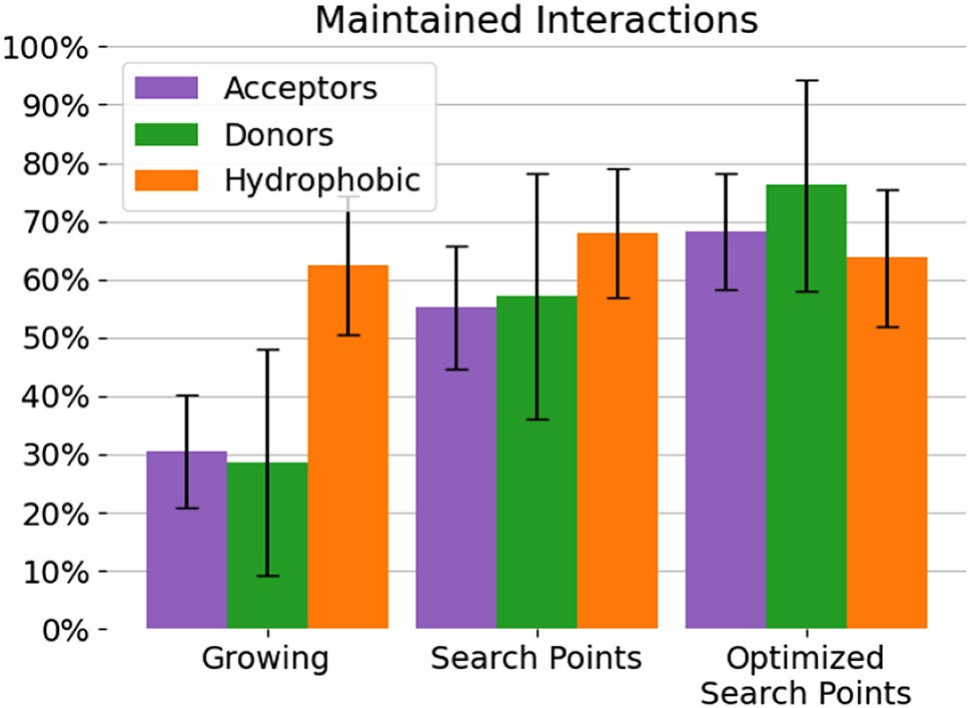
Percentage of stable interactions that could be maintained in the cross-growing set by different methods. “Growing” represents purely shape-based growing. “Search Points” and “Optimized Search Points” represent growing with search points with or without subsequent restrained JAMDA optimization. The y-axis denotes what percentage of input interactions could be maintained during growing. The three most prominent interaction types are color coded according to the legend. The error bars are 95% confidence intervals

In general, the pose generation performance of growings with search points was better than for those without ($77.0\pm 5.2\%$ vs. $64.3\pm 5.9\%$, see Sect. 4 of the Supplementary Information). It is, however, more meaningful to evaluate the interaction geometries themselves that growings with search points produce. Figure 1 shows what percentage of interactions could be maintained by the grown poses. The purely shape-based growing conserved $62.4\pm 12.0\%$ of the hydrophobic interactions, but had significant difficulties maintaining hydrogen bond acceptor and donor interactions at $30.6\pm 9.8\%$ and $28.6\pm 19.4\%$, respectively. Shape-based growing had no way of telling where to place hydrogen bond interactions because it did not receive any search point information and the RVM descriptor does not contain any electrostatic information [18].

FastGrow using search points and FastGrow using restrained JAMDA optimization as well as search points both outperformed purely shape-based growing in conserving acceptors. FastGrow using search points had a success rate of $55.3\pm 10.6\%$ and FastGrow using search points with optimization had a success rate of $68.2\pm 9.9\%$. Furthermore, hydrogen bond donor functionalities, as the most geometrically constrained interactions in this set, seemed to profit the most from restrained JAMDA optimization after a search point query with $76.2\pm 18.2\%$ of them being conserved. The upper bound to maintaining any interactions was the performance of the pose generation. Search points with JAMDA optimization showed a very similar ability to maintain interactions as the pose generation performance.

### Water replacement

**Fig. 2:**
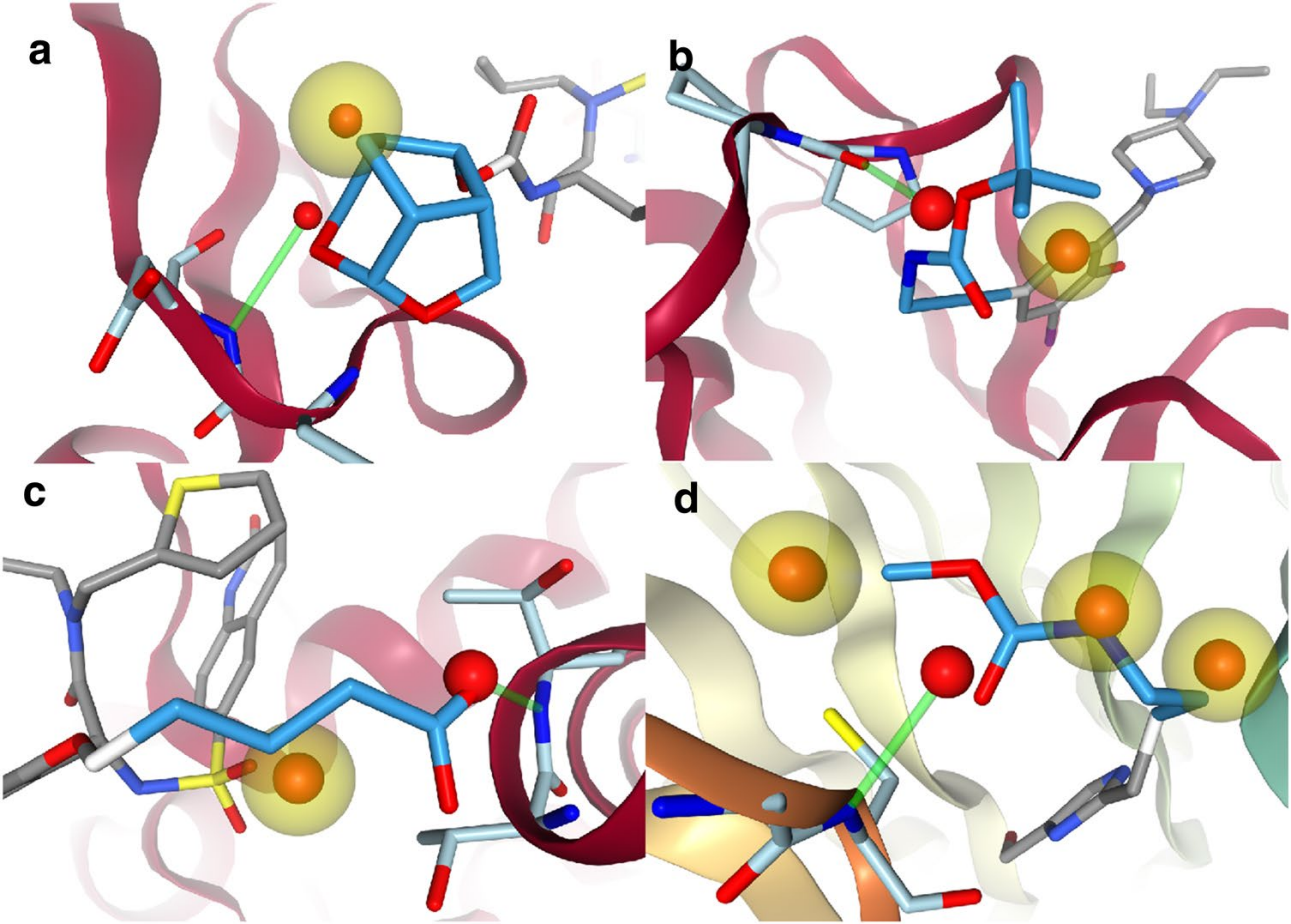
Water replacement test cases that were extracted from the cross-growing set. The replaced waters are shown with the interactions that were used in the query to replace them. Yellow spheres are hydrophobic interactions and green cylinders hydrogen bond interactions. Binding site residues are light blue. The ligands are non-native to the binding site. The darker blue part of a ligand will be grown in a water replacement test case. **a** The ligand of 5ULT in 3GI6 (Gag-Pol polyprotein). **b** The ligand of 4AGO in 4AGM (Cellular tumor antigen p53). **c** The ligand of 6MA4 in 6MA5 (O-GlcNAc transferase). **d** The ligand of 6PGA in 6PG4 (WD repeat-containing protein 5). All 3D molecule images were made with the NGL viewer[38]

Water replacement test cases could be extracted for 162 cross-growing test cases. 81 cases generated one search point for the water replacement query. 58 cases generated two search points, which implied that in these test cases two waters were replaced by the ligand to be grown. The highest number of waters replaced by a ligand was four. Figure 2 shows a few examples of the ligand to be grown overlapped with water in the binding site used for growing. While some of these waters were replaced by hydrophobic substructures, some were in positions that the ligand subsequently occupied to make directed interactions with the binding site.

**Fig. 3:**
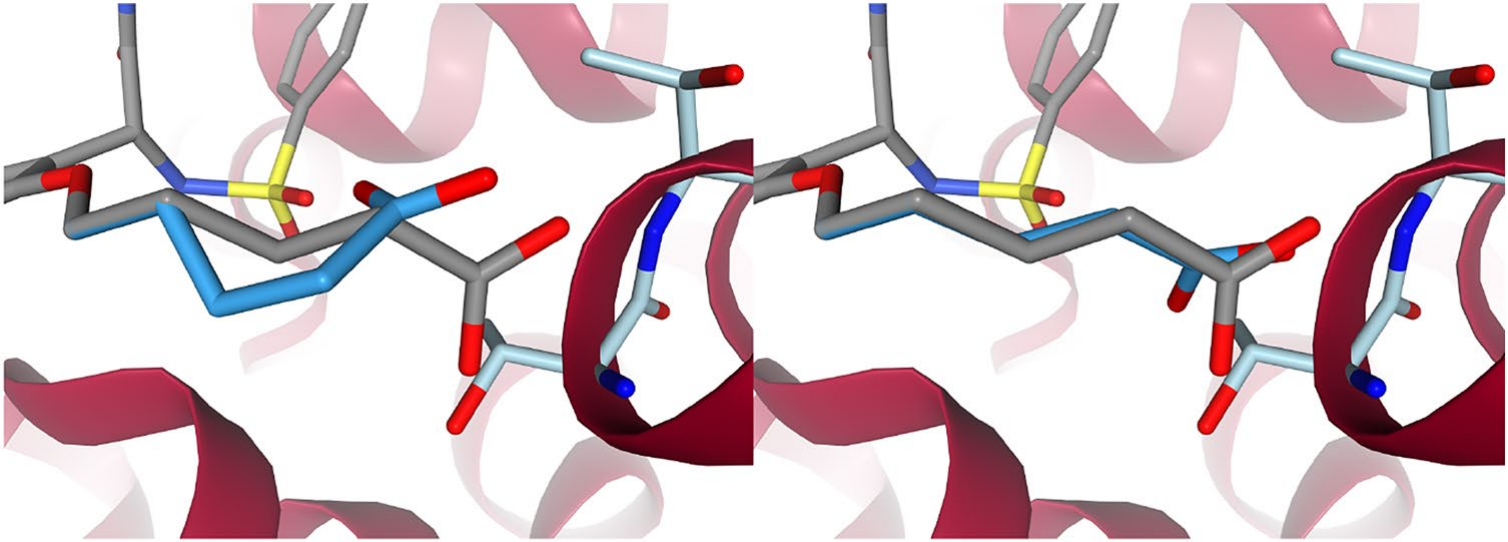
Growing with and without water replacement. The purely shape-based growing is to the left and the one using water replacement is to the right. The binding site is from PDB code: 6MA5. The ligand of 6MA4 in gray is aligned to the binding site and used as a reference for RMSD calculation. The grown ligands are in a darker blue

Although there was a difference in pose generation between purely shape-based growing with a $64.2\pm 7.4\%$ success rate and water replacement with subsequent optimization at $72.8\pm 6.8\%$, it was not significant. The somewhat small effect may in part be a symptom of the comparatively few test cases. Water replacement does not seem to confer a significant general advantage and may be more useful in specific systems. Figure 3 shows a side chain of a quinolinone-6-sulfonamide derivative crystallized in PDB code: 6MA4 being grown into 6MA5. The purely shape-based growing without water replacement did not know about any of the interactions the carboxylic acid could make with the backbone and turned it away. Using the waters in the binding site led to the side chain fully stretching out and interacting with the backbone.

### Ensemble flexibility

Ensemble test cases with at least two binding site conformations, not including the reference PDB, could be generated for 246 cross-growing test cases. Almost half of the ensemble test cases (120) contained five binding site conformations. 29 ensemble cases had only two binding site conformations. Except for five test cases, the average all atom RMSD of the ensemble binding sites to the SIENA query was between 0 and 1.5 Å distributed around a mean RMSD of 0.74 Å.

Automatically generated binding site ensembles did not show a strong statistical improvement of the pose generation performance ($69.3\pm 5.7\%$ vs. $75.2\pm 5.4\%$, see Sect. 6 of the Supplementary Information). It is well-known that ensembles tend to cause false positive hits in docking [39] and while many systems profited from ensembles, a similar amount generated poses with higher RMSDs.

**Fig. 4:**
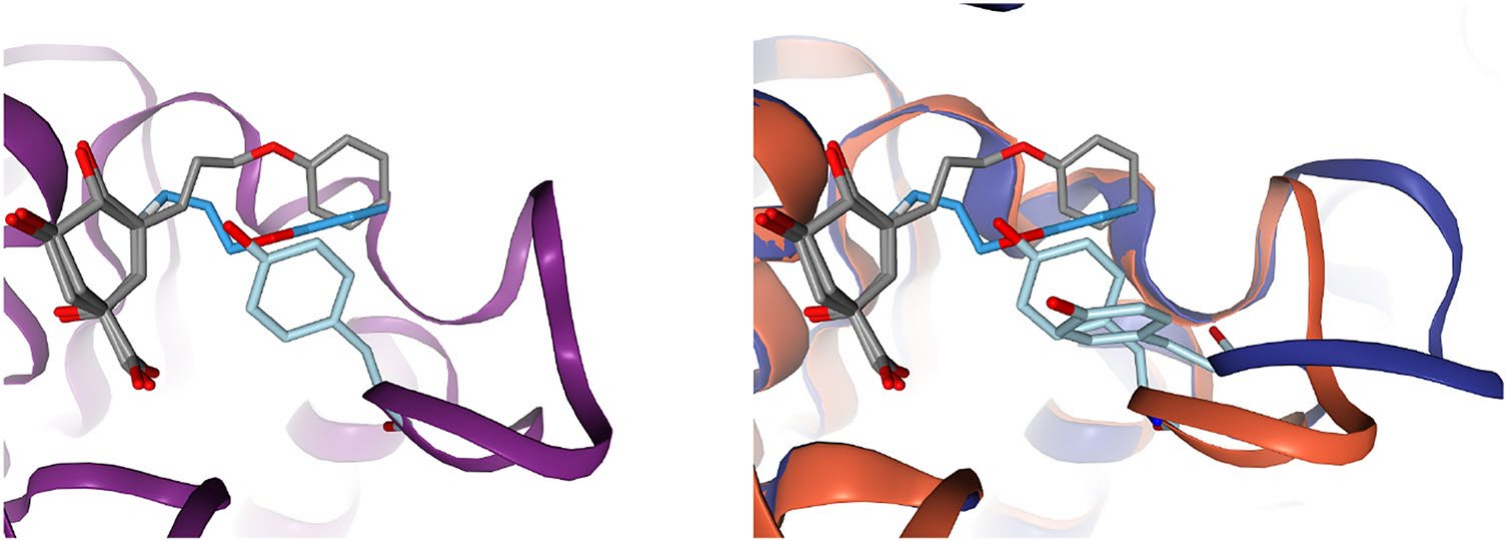
Growing in a binding site with a highly flexible loop [41]. To the left in purple is PDB code: 3N7A. To the right are 4KIU in orange and 4B6O in blue. All are structures of the 3-dehydroquinate dehydratase. The conserved TYR24 is in light blue. The ligand atoms in darker blue were grown by FastGrow in the 4KIU/4B6O ensemble, which was not possible in 3N7A alone. The reference ligand from 3N76 is in gray

When used appropriately, ensemble flexibility can make a significant difference [40]. Conformational changes of binding site residues may obstruct growing of a ligand bound in a different structure of that binding site. Figure 4 shows a shikimate precursor mimicking ligand from PDB code: 3N76 that was grown into 3N7A and an ensemble of 4B6O and 4KIU. FastGrow could not grow the phenolic substituent of 3N76 into the groove that was defined by 3N7A. It needed information about the flexibility of the loop and the movement of a conserved Tyrosine to calculate a successful pose with less than 2 Å RMSD.

### Docking comparison

The pose re-prediction performance of FastGrow was compared to DOCK [20] on the cross-growing set. Only test cases where both methods could generate at least one pose were included in the statistics and only the top poses compared. Anchored docking could not generate poses for 12 test cases and FastGrow could not generate poses for 5 test cases. One of these test cases overlapped, so 16 (4%) of the 425 test cases were excluded. A discussion of why molecules failed can be found in Sect. 7 of the Supplementary Information.

**Fig. 5:**
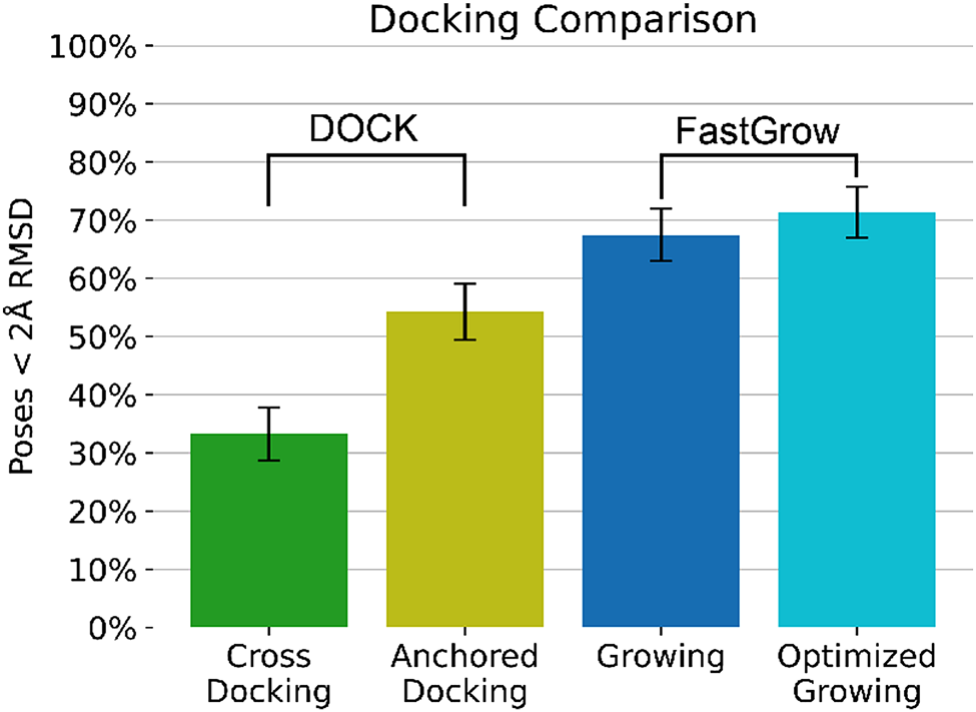
Performance comparison of DOCK in a “Cross Docking” and an “Anchored Docking” to FastGrow with or without subsequent restrained JAMDA optimization. The error bars are 95% confidence intervals

Figure 5 shows the performance of DOCK cross-docking and anchored docking versus FastGrow growing and growing with subsequent restrained JAMDA optimization. Anchored docking with a success rate of $54.3\pm 4.8\%$ outperformed cross-docking at $33.3\pm 4.6\%$, which is to be expected. Anchored docking received more input information, i.e., the core of the cross-growing test case, which significantly reduced the degrees of freedom in the system and therefore the potential for error. It is nonetheless an important point to make that using all the input information available can significantly impact the correctness of a prediction.

Both growing at $67.5\pm 4.5\%$ and growing with optimization at $71.4\pm 4.4\%$ significantly outperformed anchored docking. It was unexpected to see FastGrow outperforming anchored docking. Both systems received the same input information and had been validated for this task. The difference probably arose in how sensitive both systems were to steric clashes with the binding site. FastGrow has a comparatively high clash tolerance in the initial pose generation, which produced many poses a typical docking workflow would have rejected [18]. Restrained JAMDA optimization then resolved these clashes with minor geometry adjustments. The permissiveness of the pose generation may have been able to sample the sometimes uncomfortable fit of a non-native ligand better than a more clash sensitive system.

### DYRK1A case study

DYRK1A is one member of the Dual Specificity Tyrosine-phosphorylation-regulated Kinases. Its expression pattern suggests a role in the central nervous system [42], which supports its implication in neurodegenerative disease [43] and Down’s Syndrome [44]. Furthermore, DYRK1A is suspected to be involved in some of the pathways that lead to an increased cancer risk for individuals with Down’s Syndrome [45]. Servier and Vernalis recently published a collection of novel and highly active inhibitors for DYRK1A that were produced in a collaborative FBDD campaign in Walmsley et al. [21] and Weber et al. [22].

#### Growing a ligand from a fragment

**Fig. 6:**
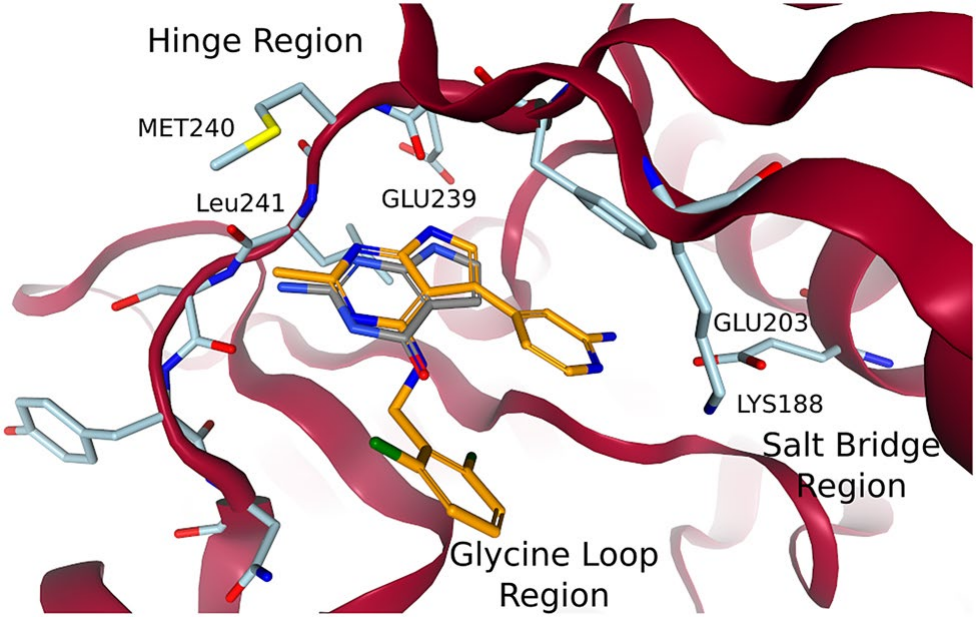
Fragment 1 and compound 34 in a structure of DYRK1A. The structure and fragment 1 coordinates in gray are from PDB code: 7A4R and compound 34 in orange comes from PDB code: 7A5N and is aligned to the binding site using SIENA [27]. The cartoon for the residues GLU160 to ASP178 was removed for clarity as it is in Walmsley et al. [21]. All 3D molecule images were made with the NGL viewer [38]

Walmsley et al. discovered fragment 1 (PDB code: 7A4R) as one of their micromolar hits (DYRK1A cKi 1.5 $\mu$M) from a fragment screening that was performed on DYRK1A using the Vernalis fragment library [21, 46, 47]. Toward the end of the publication they focused on compound 34 (PDB code: 7A5N), a nanomolar ligand (DYRK1A IC$_{50}$ 7 nM) that they described as *“[...] a potent, in vivo-tolerated, selective inhibitor of DYRK1A kinase”* [21]. Both fragment 1 and compound 34 can be seen in Fig. 6. Both fragment 1 and compound 34 exhibited canonical hinge binding [48] and compound 34 retained the central ring core of fragment 1. Compound 34 could therefore be grown from fragment 1 by exchanging the amine close to the hinge to a methyl, growing into the salt-bridge region, and using the vector defined by the carbonyl at the ring of fragment 1 to address the glycine loop. These three steps were performed with FastGrow and DOCK.

**Fig. 7:**
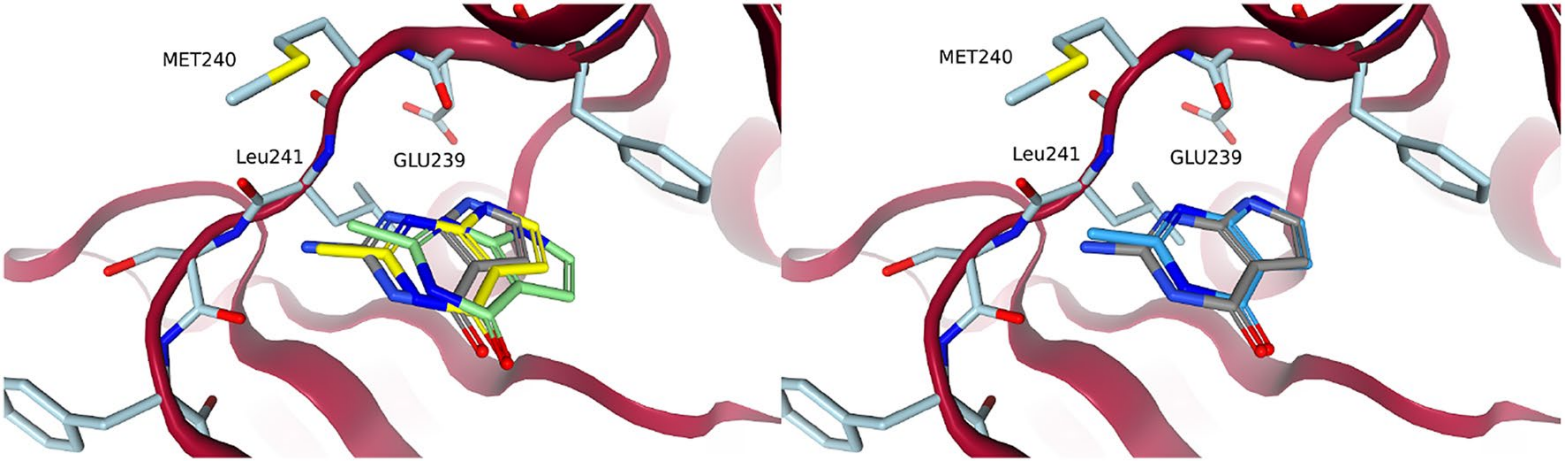
Amine to methyl exchange of fragment 1. Fragment 1 from PDB code: 7A4R is in grey. To the left are poses that were generated by DOCK cross-docking in light green and anchored docking in yellow. To the right is the pose that was generated by FastGrow in a darker blue

Exchanging the amine of fragment 1 to a methyl was an important step towards the selectivity of compound 34. The uncommon position of the LEU241 carbonyl opened up space near the hinge, which was specific to DYRK1A [21]. Computationally, the switch from amine to methyl should be trivial. Figure 7 shows the poses that were generated by DOCK and FastGrow. Both the anchored docking approach using DOCK and FastGrow generated a realistic pose for fragment 1 with a methyl. Cross-docking with DOCK, which was not constrained by the position of the core atoms, generated a pose that drifted out of the pocket. Clearly, not using the available information of the core atoms led to a poorer quality pose. Although the change in RMSD is minor, several interaction geometries shifted into the unphysical range.

**Fig. 8:**
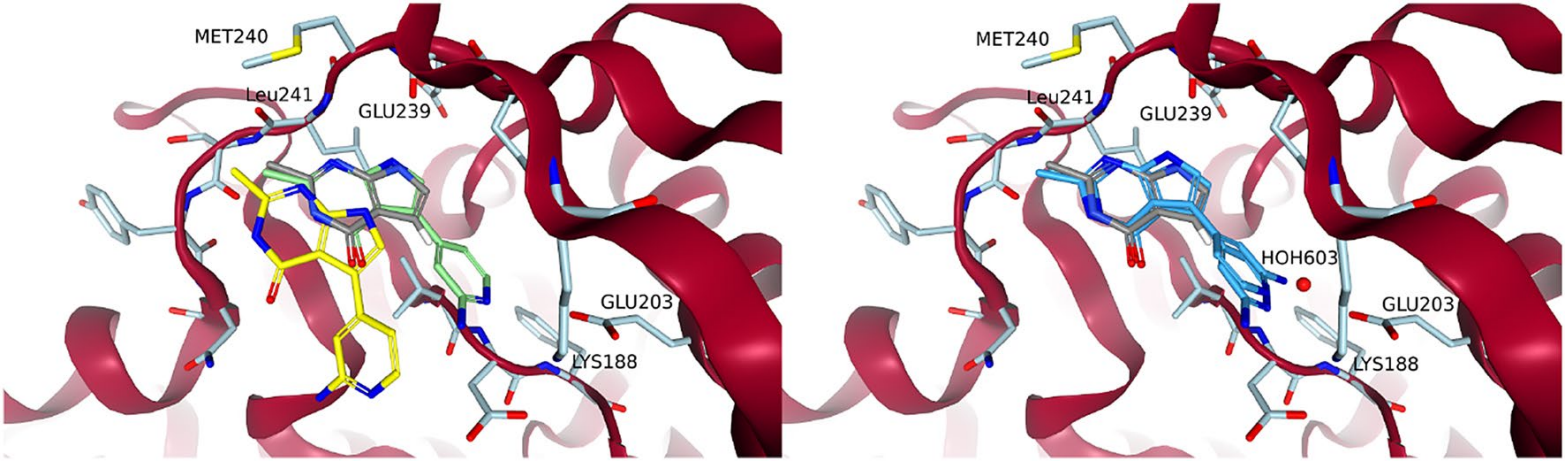
Growing the aminopyridine into the salt-bridge region. The core of methylated fragment 1 that was used as an input to FastGrow and anchored docking is in grey. To the left are the poses that were generated by DOCK cross-docking in green and anchored docking in yellow. To the right are two poses produced by FastGrow in darker blue. The pose with the amine towards GLU203 was guided by placing a search point at the position of HOH603

A hydrogen position at the pyrrole substructure of the pyrrolopyrimidine could be used to grow into the salt-bridge region. The pyridine nitrogen of the aminopyridine to be placed there was expected to interact with LYS188, while the amine was expected to interact with GLU203. Figure 8 shows the poses generated by the three methods. Using an anchored docking or a cross-docking configuration led to significantly different poses in this case. The cross-docking generated a pose very close to the eventual orientation the aminopyridine has in compound 34 (i.e., PDB code: 7A5N). Despite receiving the coordinates of the full methylated fragment 1 as an input, the anchored docking optimized its pose out of the pocket. This could have been an overreaction of the underlying scoring function and optimization to minor clashes. Cross-docking did find a good pose, proving that both the scoring function and optimization *could* support one, however some strong effect perceived by the optimization led to a poorer pose for anchored docking.

All methods initially placed the amine of the aminopyridine away from GLU203. There are structures that support this as at least a reasonable position. Weber et al. modeled the ligand of PDB code: 7AJ2 with multiple conformations one of which has an aminopyridine that points away from GLU203. Most structures reported by Walmsley et al. and Weber et al., however, were modeled so that the aminopyridine pointed toward GLU203. We could achieve a pose more consistent with the other structures by using the position of the water HOH603 to estimate a reasonable position for the amine to interact with GLU203. HOH603 was then removed as usual in the growing process. We could also use the position of the amine in a different structure to achieve the same result. A search point with the correct type at that position guided FastGrow to place the amine near GLU203.

**Fig. 9:**
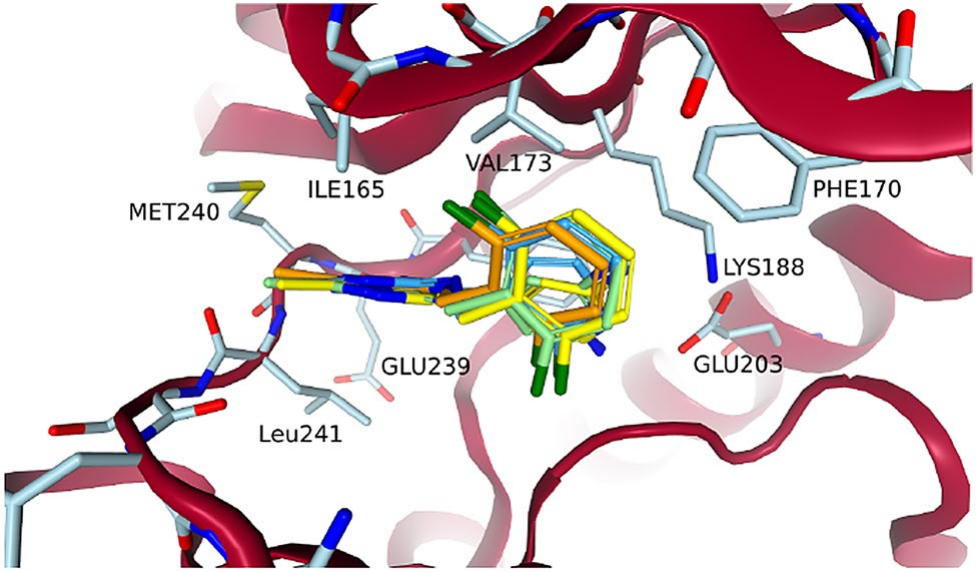
Poses of compound 34 that were grown from fragment 1 in PDB code: 7A4R. In green and yellow are the DOCK cross-docking and anchored docking poses, respectively. The FastGrow pose is in a darker blue. The compound 34 reference structure aligned to 7A4R is in orange

The last growing step involved replacing the carbonyl in the now modified fragment 1 with a difluoro-benzylamine, which interacts with the glycine loop. Figure 9 shows that all methods agreed on this step. The anchored docking, cross-docking, and FastGrow all reproduced the conformation of the difluoro-benzylamine of compound 34 in 7A5N.

Each of the three growing vectors considered above resulted in at least one series of compounds in the publication by Walmsley et al. [21]. While this case study was therefore reductive, it demonstrated the three methods: DOCK cross-docking and anchored docking, as well as FastGrow, in a practical application. Cross-docking generated good poses for the two larger modifications but exhibited unnecessary pose drift in the methylation. Anchored docking incorporated template information but seemed to have stability issues when confronted with clashes. FastGrow generated realistic poses for all three parts of the incremental growing. In one case the pose generation could be improved by using a crystallized water or external information to place a search point as guidance.

#### Fragment growing enrichment

**Fig. 10:**
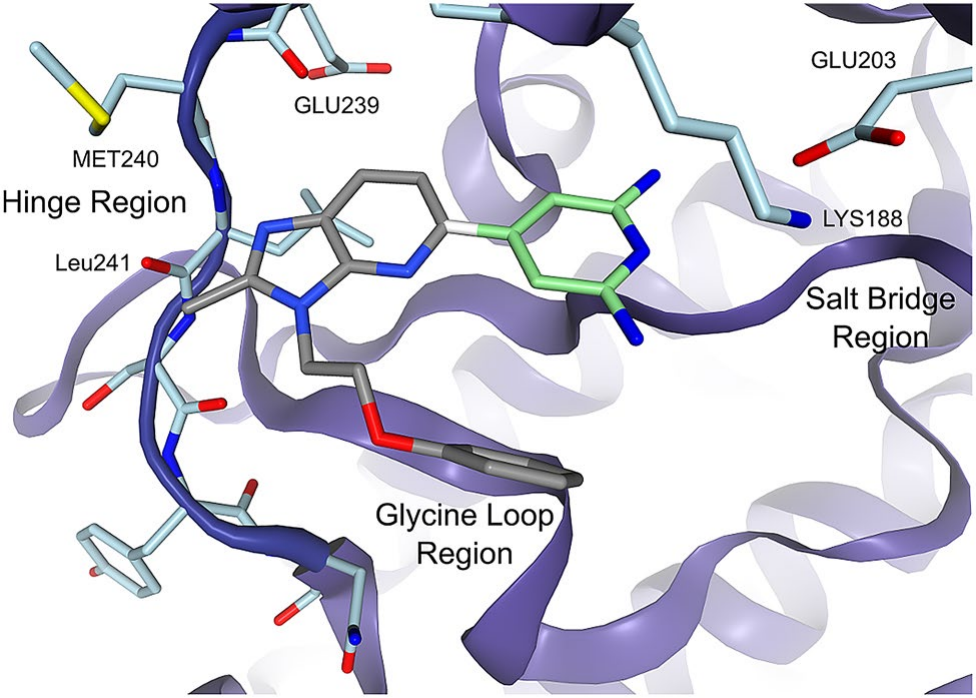
The fragment screening enrichment core generated from PDB code: 7AJW. The diaminopyridine many of the ligands in the data set have in common is in light green. The bond between the gray and the green substructures is cut and the green structure used for growing. The growing direction will therefore be towards the hinge region

A number of ligands from Walmsley et al. [21] and Weber et al. [22] had common cores that could be used to grow other ligands. The diaminopyridine moiety of the ligand in PDB code: 7AJW was used as the growing seed or core for the enrichment case study. Figure 10 shows the ligand of 7AJW with the diaminopyridine highlighted in green. Any fragment growing from this core would initially point directly to the hinge region.

Many ligands of DYRK1A, especially those from Weber et al. had a conserved diaminopyridine moiety. Filtering all diaminopyridine-containing ligands down to only the ones with affinities of less than 10 nM resulted in 77 ligands. These 77 ligands were fragmented at the bond to the diaminopyridine. The Rule of Three [35] properties of these fragments were inspected to detect outliers far outside of the property distribution. Nine fragments were discarded as outliers. There was a large group of fragments with exactly the same TPSA, which had to be downsampled to avoid biasing the screening. This lead to a collection of 40 highly active fragments. A comparison of properties before and after the filtering can be found in the Supplementary Information, Sect. 8.

**Fig. 11:**
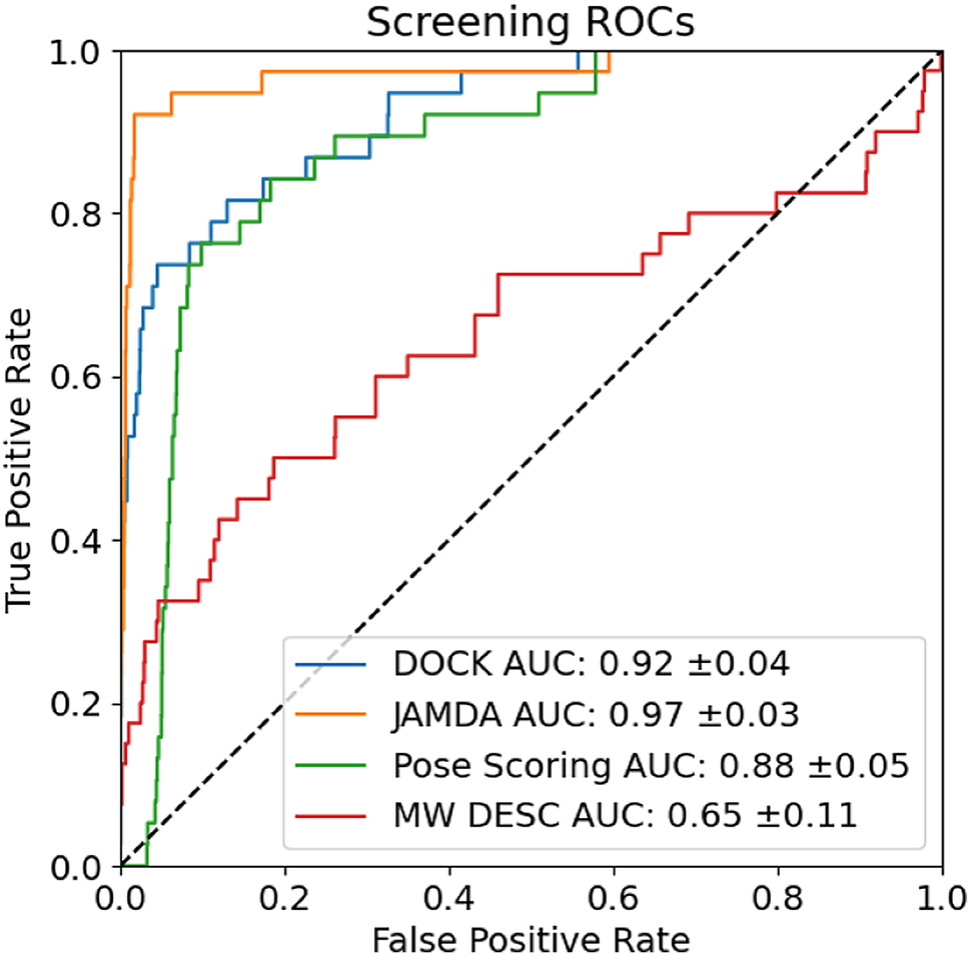
ROC curves and AUCs of DOCK anchored docking, FastGrow with JAMDA, the FastGrow pose scoring function and sorting by descending molecular weight. The annotated errors on the AUCs describe a 95% confidence interval

The set of 40 known highly active single-digit nanomolar fragments was combined with a set of generic fragments assumed to be not as active. 2653 property-matched generic fragments were generated using the BioSolveIT fragment set [34]. The fragments were property matched using “Rule of Three” properties [35]. After property matching, sorting by molecular weight was chosen as a comparison baseline. Molecular weight retained some residual discriminative power despite property matching and was therefore chosen as a comparison baseline instead of an idealized null baseline. This is discussed further in Sect. 8 of the Supplementary Information. For evaluation we used the receiver operating characteristic (ROC) and the area under the curve (AUC). The ROC curves and AUCs of the three methods (FastGrow’s pose scoring, FastGrow with restrained JAMDA optimization and anchored docking with DOCK) can be seen in Fig. 11.

All methods outperform the molecular weight baseline. The methods themselves all performed similarly and very well. Most surprising is that the FastGrow pose scoring function, which was never parametrized or evaluated for this purpose, performed almost on par with the other more sophisticated scoring functions. The FastGrow pose scoring function is made up of three terms: filled volume, number of close contacts, and clash [18]. Clash is the only term it shares in common with the other two scoring functions and therefore must be the driving force behind the general high performance. To substantiate this we repeated the experiment with all terms of the pose scoring function set to zero except for clash. Figure S7 shows that the pose scoring function with just clash performed comparably to the other scoring functions. 3D clash, and by extension shape complementarity, seemed to be a very discriminative property in this particular system, which leads to the high performance of all three of these methods.

Table 2 shows the time it takes for each of the three methods to generate poses for all fragments in the enrichment screening and score them. There was a factor five difference in speed between DOCK anchored docking and FastGrow with restrained JAMDA optimization. There was a factor 500 difference in speed between anchored docking and using FastGrow’s pose scoring function. Of the 2 s FastGrow and its pose scoring function spent screening, half was spent in the input-output operations of extracting 2693 fragments from the screening database and writing the hits. The hundred-fold increase in runtime between the simple FastGrow pose scoring function, which performed competitively in this case study, and the more sophisticated scoring functions was disproportional to the apparent gain in performance.

**Table 2:** Runtimes of fragment growing enrichment for the three methods

Method	Screening time [s]	Runtime per fragment [ms]
FastGrow + Pose scoring	2	0.70
FastGrow + JAMDA	226	84.04
Anchored docking	1179	437.75

Screening time denotes a full screening with all 2693 fragments in seconds. Runtime per fragment is the arithmetic mean over all fragment runtimes in milliseconds. Machine specifications can be found in Sect. 1 of the Supplementary Information

## Conclusions

FastGrow is a novel and very fast approach for structure-based fragment growing. It achieved competitive pose re-prediction performance and enrichment compared to other well-known docking workflows but significantly outperformed these in speed. FastGrow can be used not only to screen larger collections of fragments, but also to reveal trends in fragment types and poses that are not visible in shorter hit lists. Besides the purely shape-based growing, FastGrow has been equipped with optional pharmacophore-like constraints, which can also be used to displace water molecules. Moreover, it may read in and work with an ensemble of protein structures to describe the flexibility of a target.

Our validation showed that we could maintain important interactions during growing. Success varied depending on the type of interaction, but modeling interactions as pharmacophore-like search points generally led to an improvement in pose re-prediction. Especially the more geometrically constrained interactions profited from restrained JAMDA optimization. We validated displacing waters by using those visible in crystal structures as hints for potential interactions, which improved pose generation in some scenarios. The ubiquitous tendency towards false positives in ensemble flexibility approaches meant that we could not find a pronounced statistical improvement when using ensemble flexibility. However, it was shown that some systems profited from or even required multiple structures to describe binding properly.

A growing case study on a DYRK1A FBDD campaign demonstrated the advantages and shortcomings of both DOCK and FastGrow in iterative growing. Using the position of a fixed fragment as input had clear advantages in both the statistical evaluation on the cross-growing set, as well as the case study. Anchored docking with DOCK encountered stability issues in the case study, which could not happen with FastGrow, due to its restrained optimization and the inherent stability of the JAMDA scoring function [19]. We could also demonstrate FastGrow’s pose generation being guided by including external interaction information in the query.

The enrichment case study on DYRK1A demonstrated a scenario that could largely be solved by clash or in other words shape complementarity. A general statistical evaluation should however also include examples that require electrostatic complementarity in addition to shape complementarity. Building up a balanced dataset of such cases without biasing it by the methods to be evaluated is a significant challenge and beyond the scope of this work. A more general analysis will be necessary to address the lingering questions of how to incorporate shape and interactions into a scoring function of appropriate complexity.

As is often the case, the inclusion of external and inferred information is generally more successful than building a generalized model that encodes this information. It is for this reason we have focused on interactive, intuitive and quickly iterable approaches in FastGrow. Its ability to generate new ligands is comparable to established approaches and it would be interesting to see its sampling improved and compared to newer machine learning based generative models. FastGrow is already in use for current projects at Servier, as well as other organizations, and we hope it will enable further interesting results in the near future.

## Supplementary Information

Below is the link to the electronic supplementary material.

Supplementary file 1 (pdf 582 KB)

Supplementary file 2 (zip 344 KB)

Supplementary file 3 (zip 300339 KB)

## Data Availability

All data generated or analysed during this study is included in the supplementary information files.

## Software availability

The FastGrow web server is available at https://fastgrow.plus/. The code of the FastGrow web server is available at https://github.com/rareylab/fast_grow_server. Automation scripts for DOCK execution are available at https://github.com/rareylab/dock_scripts. The core functionality is a part of the fragment-based “Inspirator Mode” in BioSolveIT’s SeeSAR modeling package [49].
