# Supplementary material for: FastGrow: on-the-fly growing and its application to DYRK1A
Source: J Comput Aided Mol Des. 2022 Aug 22;36(9):639–51. doi: 10.1007/s10822-022-00469-y (PMC9512872; doi:10.1007/s10822-022-00469-y)
Supplement: Supplementary file 1 — (pdf 582 KB) [file 10822_2022_469_MOESM1_ESM.pdf]

Supplementary Information: FastGrow: On-the-Fly Growing and its Application to DYRK1A

Patrick Penner[1], Virginie Martiny[2], Louis Bellmann[1], Florian Flachsenberg[1,4], Marcus Gastreich[3], Isabelle Theret[2], Christophe Meyer[2] and Matthias Rarey[1*]

[1]ZBH - Center for Bioinformatics, Universität Hamburg, Bundesstr. 43, 20146, Hamburg, Germany.
[2]Institut de Recherches Servier, 125 Chemin de Ronde, 78290, Croissy-sur-Seine, France.
[3]BioSolveIT GmbH, An der Ziegelei 79, 53757, Sankt Augustin, Germany.
[4]Current Address: BioSolveIT GmbH, An der Ziegelei 79, 53757, Sankt Augustin, Germany.

*Corresponding author(s). E-mail(s): matthias.rarey@uni-hamburg.de;

1 Machine Specifications

All calculations were performed on a machine with the following specifications:

OS	OpenSUSE Leap 15
CPU	Intel(R) Core(TM) i5-8500 CPU @ 3.00GHz
RAM	16 GB
Harddrive	Locally Mounted SATA 6.0 Gb/s HDD

Table S1 Specifications of the machine used for all calculations

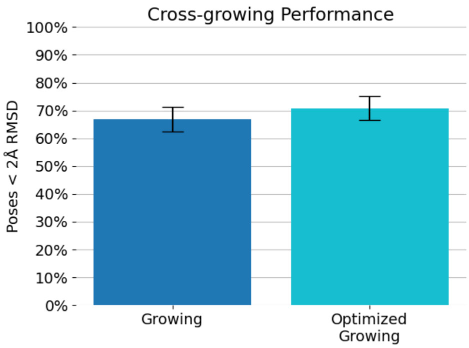

Fig. S1 Pose re-prediction performance on the cross-growing set for the shape-based "Growing" and restrained JAMDA "Optimization". The error bars are 95% confidence intervals

2 Statistical Methods

Performance of pose re-prediction was expressed as a percentage of poses a method generates that have an RMSD of less than 2Å. The threshold of less than 2Å made this a binary classification. A binary classification and its results, which generate a binomial distribution, approaches a normal distribution. Within certain bounds we could therefore apply parametric methods[1]. A 95% confidence interval in a normal distribution covers 1.96 standard deviations. All pose re-prediction experiments were tested whether they could be approximated by a normal distribution. The confidence interval could then be calculated using the individual standard deviations.

Area under the curve (AUCs) of the receiver operating characteristics (ROC) curves were used to evaluate the enrichment performance in the fragment growing enrichment validation. ROC curves and their AUCs are very specific but established evaluations of enrichment screening[2]. 95% confidence intervals for AUCs were calculated by bootstrap re-sampling[3]. This too can now be considered established in literature by multiple high-profile publications[4, 5].

3 Performance on the Cross-growing Set

Figure S1 shows FastGrow pose re-prediction performance on the cross-growing set using just the shape-based growing and restrained JAMDA optimization. The main publication states movement in the core atoms was to blame for inconclusive performance differences. Core atoms are not actually considered in any RMSD used for validation, but small movements in the core atoms may have had a large impact on the fragment atoms. Especially changes in angles may have cascaded into large RMSDs.

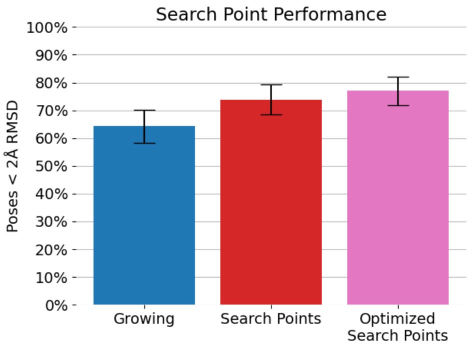

Fig. S2 Pose re-prediction performance on the subset of cross-growing systems with stable interactions used as search points. To the left is the shape-based "Growing". The middle bar represents shape-based growing using "Search Points". The rightmost bar represents growing with search points and subsequent restrained JAMDA optimization. The error bars are 95% confidence intervals

4 Maintaining Interactions

In general the pose generation performance of growings with search points was better than those without. On this subset of the cross-growing set purely shape-based growing achieved 64.3% (95% CI [58.4%, 70.2%]) in re-predicting ligand poses. The addition of search points improved on this with a 73.8% success rate (95% CI [68.4%, 79.2%]). It took subsequent restrained JAMDA optimization to significantly improve poses with a success rate of 77.0% (95% CI [71.8%, 82.2%]).

Search points noticeably moved poses closer to the crystal pose of the reference ligand they were taken from. The main validation question in this subset, however, was whether the interactions the search points were generated from could actually maintained. All three methods performed quite similarly when it came to hydrophobic interactions. These are the least geometric interactions and are probably most determined by pose quality. The main publication discussed the more relevant differences in the quality of hydrogen bond interactions.

5 Water Replacement

Figure S3 shows the performance of FastGrow on the water replacement subset of the cross-growing set for purely shape-based growing, using the water replacement search points and performing restrained JAMDA optimization after using the water replacement search points. None of the differences had well separated confidence intervals.

Performing restrained JAMDA optimization after using the water replacement search points achieved a very similar performance as using optimization and search points in the interaction maintenance validation, further supporting those results. It was interesting to see how search points alone seemed to

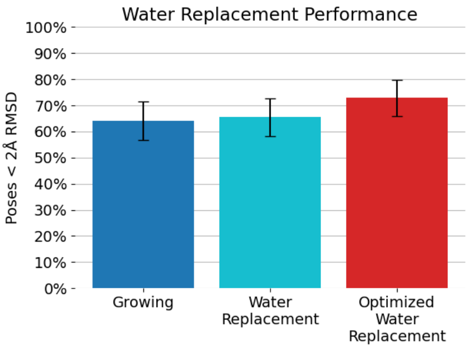

Fig. S3 Performance of water replacement by search points on a subset of the cross-growing set. To the left is the shape-based "Growing". The middle bar shows the performance of placing search points on water positions that are replaced by the ligand to be grown. The right bar represents water replacement by search points with restrained JAMDA optimization. The error bars are 95% confidence intervals

perform worse in this situation. A possible explanation is that because these search points had less to do with the ligand to be grown than in the interaction maintenance section the ligand was initially forced into somewhat more uncomfortable poses. These were then relaxed using JAMDA optimization and lead to better poses.

6 Ensemble Flexibility

Figure S4 shows the performance of growing with ensembles in comparison to growing with the single input protein, which was used as a query to build the ensembles. Shape-based growing with one input binding site managed to successfully generate poses for 69.3% (95% CI [63.5%, 75.0%]) of the ensemble test cases. Neither growing with ensembles at 72.8% (95% CI [67.2%, 78.3%]) of poses less than 2 Å nor growing with ensembles with optimization at 75.2% (95% CI [69.8%, 80.6%]) significantly outperformed shape-based growing. Both were slightly better but none of the error bars could be considered separated.

At first glance the results seemed to suggest that growing with ensembles does not lead to significantly better results. 202 of 245 test cases did not cross the less than 2 Å threshold in either direction when comparing growing with one input protein and growing with ensembles. 26 test cases that failed with just one input protein are successful when growing with an ensemble. 17 test cases that were successful with just one input protein, failed with ensembles. Although the effect is small more cases are improved with ensemble flexibility.

The phenomenon that some results get worse using ensembles is usually attributed to the tolerance introduced by multiple proteins leading to false positive poses[6]. This will no doubt also be a contributing factor in this validation. The largest fraction of test cases however does not change significantly due to ensembles. Both of these points highlight the fact that these ensembles are generated automatically and with only minimal curation in the form of

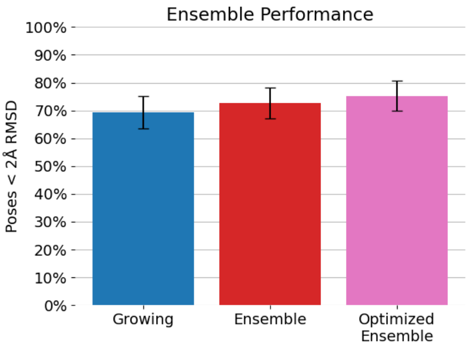

Fig. S4 Performance of ensemble growing on a subset of the cross-growing set. To the left is shape-based "Growing" with one input binding site. In the middle is the "Ensemble" growing, which uses a binding site ensemble generated SIENA. The right-most bar represents ensemble growing with subsequent restrained JAMDA optimization. The error bars are 95% confidence intervals

an all atom RMSD clustering. This is bound to introduce unnecessary noise. It is, however, difficult to build a large number of curated ensembles by hand without introducing bias, which is why we chose an automated procedure.

7 Docking and Growing failures

The performance of DOCK on the cross-growing set was lower than expected. Allen et al. determined the average cross-docking success rate for ligands in non-native pocket to be 50.8% using the protocol replicated here[7]. There were several reasons for this difference in performance. One of the most impactful is that no physico-chemical filters, such as the "Rule of Five"[8], were applied to the full ligands when generating the cross-growing test cases. The fragments had to be "Rule of Three"[9] compliant but the ligands were often significantly more complex than either set of property rules would allow. The anchored docking which replicated the fixed-anchor docking (FAD) protocol from the same publication seemed to be more in line with performance expectations.

All cross-growing test cases that failed in docking produced the error message: "Could not complete growth. Confirm that the grid box is large enough to contain the ligand, and try increasing max_orientations". Both of the suggested fixes were attempted. Manual verification showed that the ligand to be grown could be fit into docking box. The *max_orientations* parameter was consecutively increased by several orders of magnitude, unfortunately to no effect.

The error message appeared to describe a spatial sampling issue. Due to the fact that cross-growing test cases attempt to grow ligands into pockets they were not crystallized in, which means the binding sites may not have been in an ideal conformation for that ligand, these errors were to be expected. This effect was probably the cause of some of the FastGrow failures as well. One of the FastGrow failures, growing the ligand from PDB code: 5J20 into PDB

code: 5J64, could be solved by generating more conformations when creating the fragment databases. This failure was clearly due to undersampling of the conformational space of the ligand. 96% of test cases were, however, unaffected, which still guaranteed a sufficient statistical sample for the comparison to docking.

Allen et al. solved this problem by attempting to minimize a foreign ligand, in their case in a cross-docking scenario, in the binding site and discarding it if it changed by more than an RMSD of 2Å[7]. We chose not to do this because making decisions based on minimization may introduce bias, especially if the same or a similar minimizer is used in the evaluation as well. In their paper[7] they recognize that there are different ways to approach this problem and we hope they will not begrudge us the decision against involving minimization.

8 Property Matching in Detail

Property matching was implemented as a simple procedure that limited the active and generic fragments to a common min-max range with the "Rule of Three" properties[9]. This is a far simpler procedure than implemented for example by Stein et al.[5] and mostly reflects the limited amount of data available. More complex methods to fit distributions of properties to each other eliminated either too many actives or generic fragments for the data set to be useful.

Figure S5 shows the performance of sorting by "Rule of Three" properties as ROC curves. Only the sorting direction, ascending or descending by value, that performed better was considered for each property. The discrete properties "Lipinski Acceptors", "Lipinski Donors" and "Number of Rotatable Bonds(NROT)" were not well suited to evaluation by ROC curves. The variance present for these properties in Figure S5 is generated by randomizing the order of the rows within the discrete categories of these variables, for example "Lipinski Acceptor" counts of 1 to 5. There is actually only one value for "Lipinski Donor" counts in the data set which is zero. "Topological Polar Surface Area (TPSA)" was also an almost discrete value in this context. TPSA is calculated by summing up polar surface area contributions from fragments[10]. Because we calculated TPSA for fragments, which were comparatively small, the variance was small as well, which led to an almost categorical distribution. Molecular weight is the best performing continuous "Rule of Three" property and was used as the baseline.

The residual performance of the "Rule of Three" properties was a function of the different distribution of those properties in the active and generic set. Figure S6 shows molecular weight histograms for the active and generic fragments. Both histograms span the same range of values for molecular weight but have noticeably different distributions. The decoy distribution skews slightly towards lower molecular weights and the active distribution skews slightly to higher molecular weights. This results in the residual 0.65 ± 0.11 AUC when the full fragment set was sorted by descending molecular weight.

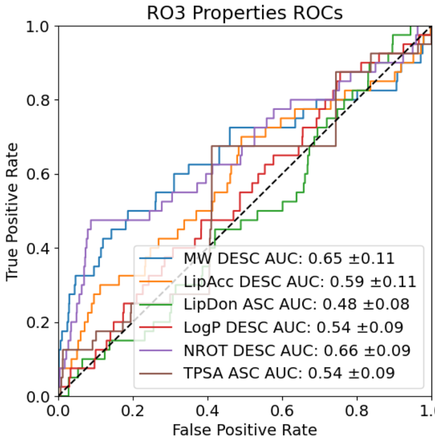

Fig. S5 ROC curves and AUCs of sorting by "Rule of Three" properties. Only the better sort order, ascending or descending value, by AUC is shown. The annotated errors on the AUCs describe a 95% confidence interval

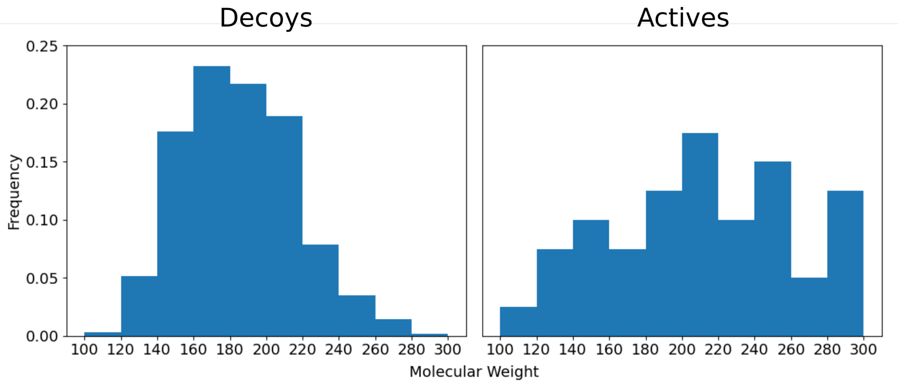

Fig. S6 Molecular weight histograms for the active and generic/decoy fragments of the fragment growing enrichment. Both histograms have the same normalized y-axis

9 Clash Experiments in Fragment Growing Enrichment

Figure S7 shows the ROC curves and AUCs of the enrichment case study including a repeat screening with the pose scoring function that only used its clash term labeled "Only Clash". The pose scoring function with all terms and with only the clash term perform comparably in this scenarios. Only clash performs slightly better than the full pose scoring function which was never parametrized for this task. This clearly suggests that clash is a very discriminative property in this scenario.

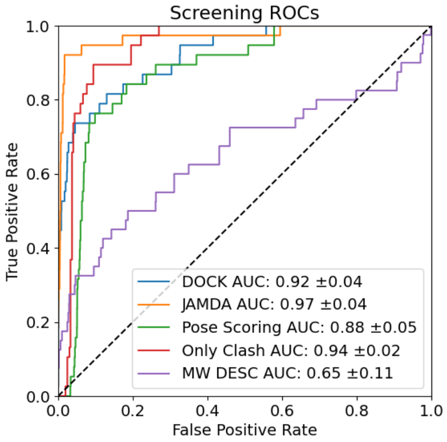

Fig. S7 ROC curves and AUCs for the DYRK1A enrichment screening. Included is the repeated screening using the FastGrow pose scoring function with only the clash term enabled. The annotated errors on the AUCs describe a 95% confidence interval

References

[1] Box, G.E.P., Hunter, J.S., Hunter, W.G.: Statistics for experimenters: Design, innovation, and discovery, 2nd edn., p. 633. John Wiley & Sons, Inc. (2005)

[2] Huang, N., Shoichet, B.K., Irwin, J.J.: Benchmarking sets for molecular docking. J. Med. Chem. **49**(23), 6789–6801 (2006). https://doi.org/10.1021/jm0608356

[3] Efron, B.: Bootstrap methods: Another look at the jackknife. Ann. Statist. **7**(1), 1–26 (1979). https://doi.org/10.1214/aos/1176344552

[4] Su, M., Yang, Q., Du, Y., Feng, G., Liu, Z., Li, Y., Wang, R.: Comparative assessment of scoring functions: The casf-2016 update. J. Chem. Inf. Model. **59**, 895–913 (2019). https://doi.org/10.1021/acs.jcim.8b00545

[5] Stein, R.M., Yang, Y., Balius, T.E., O'Meara, M.J., Lyu, J., Young, J., Tang, K., Shoichet, B.K., Irwin, J.J.: Property-unmatched decoys in docking benchmarks. J. Chem. Inf. Model. **61**, 699–714 (2021). https://doi.org/10.1021/acs.jcim.0c00598

[6] Amaro, R.E., Baudry, J., Chodera, J., Özlem Demir, McCammon, J.A., Miao, Y., Smith, J.C.: Ensemble docking in drug discovery. Biophys. J. **114**, 2271–2278 (2018). https://doi.org/10.1016/j.bpj.2018.02.038

[7] Allen, W.J., Balius, T.E., Mukherjee, S., Brozell, S.R., Moustakas, D.T., Lang, P.T., Case, D.A., Kuntz, I.D., Rizzo, R.C.: DOCK 6: Impact of

new features and current docking performance. J Comput Chem **36**(15), 1132–1156 (2015). https://doi.org/10.1002/jcc.23905

[8] Lipinski, C.A., Lombardo, F., Dominy, B.W., Feeney, P.J.: Experimental and computational approaches to estimate solubility and permeability in drug discovery and development settings. Adv Drug Deliver Rev **46**, 3–26 (2001). https://doi.org/10.1016/S0169-409X(00)00129-0

[9] Congreve, M., Carr, R., Murray, C., Jhoti, H.: A 'Rule of Three' for fragment-based lead discovery? Drug Discov Today **8**, 876–877 (2003). https://doi.org/10.1016/S1359-6446(03)02831-9

[10] Ertl, P., Rohde, B., Selzer, P.: Fast calculation of molecular polar surface area as a sum of fragment-based contributions and its application to the prediction of drug transport properties. J. Med. Chem. **43**, 3714–3717 (2000). https://doi.org/10.1021/jm000942e
